# The role of Substance P in the defense line of the respiratory tract and neurological manifestations post COVID-19 infection

**DOI:** 10.3389/fneur.2023.1052811

**Published:** 2023-03-06

**Authors:** Riffat Mehboob, Peter Oehme, Gerhard Pfaff

**Affiliations:** ^1^Lahore Medical Research Center and LMRC Laboratories, LLP, Lahore, Pakistan; ^2^Retired, Berlin, Germany; ^3^Department of Chemistry, Technical University Darmstadt, Darmstadt, Germany

**Keywords:** Substance P, COVID-19, lung inflammation, respiratory disorders, cytokine storming

## Abstract

Substance P (SP) has been a great interest for scientists due to its unique properties and involvement in various physiological and pathological phenomenon. It took almost a century for the current understanding of this peptide so far. Its role in brain and gut were initially discussed and later on it was widely studied and observed in cardiovascular system, asthma, traumatic brain injury, immune response, vasodilation, behavior, inflammation, arthritis, cancer, airway hyper responsiveness and respiratory disorders. Involvement of SP in sudden perinatal death and COVID-19 has also been discussed which shed light on its vital role in respiratory rhythm regulation and initiation of cytokine storming in COVID-19. This article will provide a comprehensive overview of the researches done to understand the basic functions and involvement of SP in different processes of cell and its association with various diseases. This article describes the historical and scientific journey of SP from its discovery until today, including its future perspectives.

## 1. Historical background

In the years 1930/31, the Swedish postgraduate student Ulf Svante von Euler (1905–1983, Nobel Prize for Physiology or Medicine 1970) isolated in the laboratory of Henry Hallett Dale (1875–1968, Nobel Prize for Physiology or Medicine 1936) in London a biological active extract from the intestine of animals (1, 2). For pharmacological studies, this extract was available as a “stable dry powder.” The P from the word “powder” was used to identify the substance and has remained part of the name “Substance P” (SP) until today. The peptide chemical group of Susan E. Leeman isolated the substance from the hypothalamus and in 1971 determined the structure to be an undecapeptide with the sequence Arg–Pro–Lys–Pro–Gln–Gln–Phe–Phe–Gly–Leu–Met–NH2 (3). The total synthesis was also performed by the Leeman group (4).

In 1976 was a special year for SP researchers. The meanwhile world-famous physiologist and pharmacologist U. S. von Euler had invited to a Nobel Symposium in Stockholm. The symposium covered the state of knowledge of SP at that time: history, chemistry, mechanisms, distribution, and pharmacology. One focus was on the effect of SP on sensory nerve endings and pain. Pioneering work on this had been done by Fred Lembeck (1922–2014), who investigated the effect of SP on afferent systems as early as 1953. His paper in Stockholm (5), and other papers, confirmed the hypothesis that SP is a transmitter in primarily sensory afferent neurons and plays an important role in the pain process. Peter Oehme, one of the authors of this contribution, together with Ulf Svante von Euler at the Nobel Symposium 1976.

**Figure 1:**
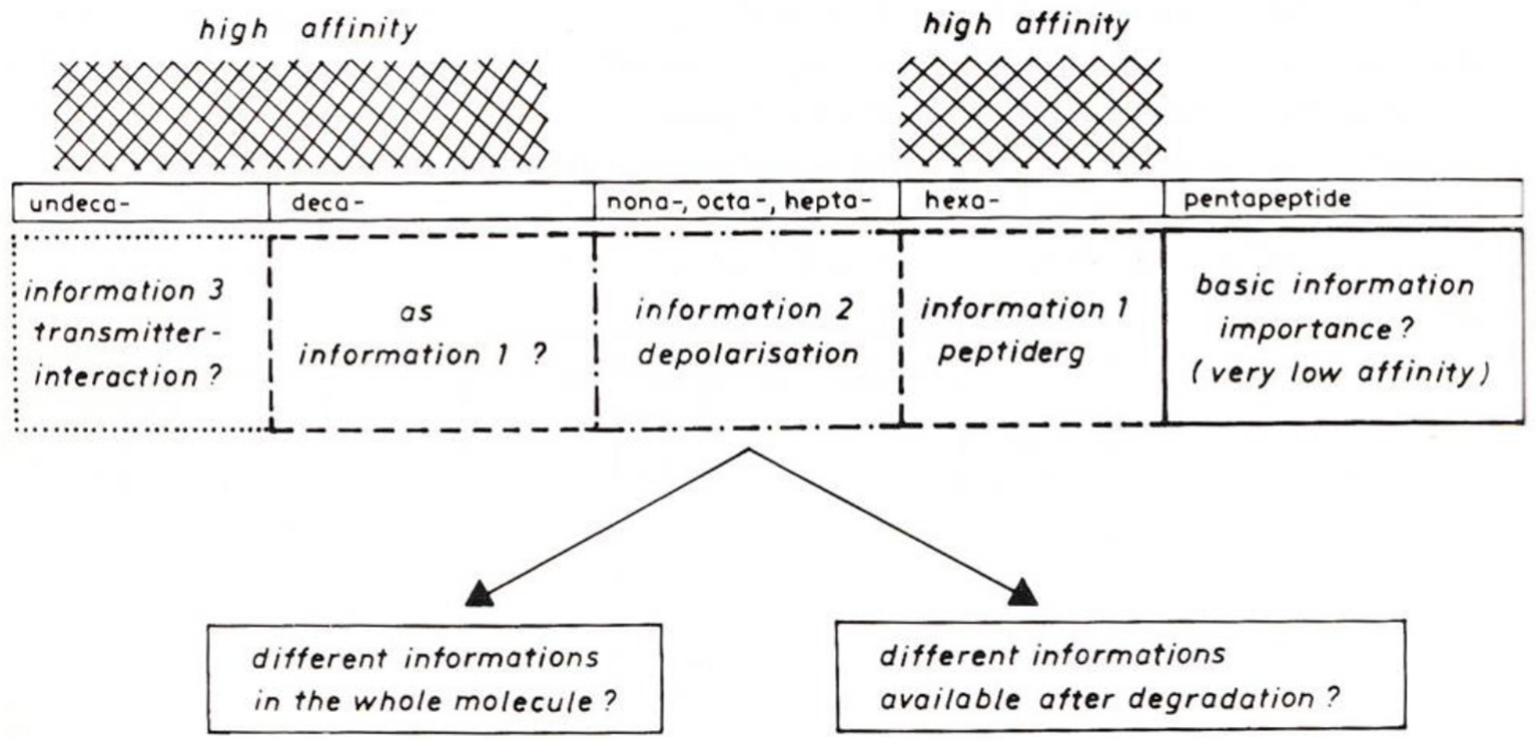
Model for different information in Substance P sequences (8).

Two papers at the symposium concerned the effect of SP on tracheobronchial tissue (6, 7). In these, the presence of SP was demonstrated in both nerve fibers and endocrine cells of guinea pig tracheobronchial tissue. At the same time, a strong effect on bronchial tone was found for SP, *in vivo* as well as *in vitro*. The effect observed was 45 times stronger than the effect of histamine. In his paper, Peter Oehme hypothesized that different information is encoded in the SP molecule (8): a direct effect on smooth muscle, sensory nerves, *etc.*, and an indirect effect through modulation of other transmitter systems, e.g., acetylcholine. For both effects, different parts of the SP sequence were discussed by Oehme (Figure 1).

## 2. Pharmacological actions of Substance P

### 2.1. Pain threshold

After returning from Stockholm, Peter Oehme started investigations on the action of SP on pain threshold in the Institute for Drug Research (IWF) of the Academy of Sciences in Berlin-Friedrichsfelde, which he founded in 1976. His investigations using the hot plate technique on mice yielded surprising results (9). It was shown that the SP effect depends on the initial condition of the test animals. It was found that SP has an analgesic effect on mice with a short reaction time to pain stimuli. On mice with a long reaction time, SP has a hyperalgesic effect. Both lead to a normalization of reaction time. Subsequent studies from the Oehme group revealed that the analgesic effect component is assigned to the C-terminal SP domain, whereas the hyperalgesic effect component is assigned to the N-terminal SP domain (10). This dual effect of SP was in accordance with the model presented by Oehme at the Stockholm SP Congress in 1976.

### 2.2. Stress reactions

Interesting findings also followed from the studies on “SP-action on behavior” carried out jointly by Oehme and Karl Hecht's group. In a series of stress models (immobilization, noise, electric footshocks, etc.), it was found in rats that SP is able to normalize the disturbances such as “decrease in learning,” “loss of deep sleep and REM sleep,” “increase in blood pressure and heart rate” (11, 12). Clinical studies conducted by Karl Hecht's group on patients with stress-induced sleep disorders with nasal SP application also showed positive results. Overall, it appeared that the N-terminus was relevant for the anti-stress effect, whereas the C-terminus was relevant for the acute effects, such as spasmogenic effect. Therefore, the term “regulatory peptide” (Regulide) was proposed for SP by Oehme and Hecht (12).

### 2.3. Pharmacological effects on chromaffin cells

Since there is an increase of catecholamines in plasma under stress, the interaction of SP with the aminergic system was investigated by the Oehme group. In adrenal slices, which contain chromaffin cells as well as endings of the splanchnic nerve, the electrically stimulated release of acetylcholine was investigated in addition to the release of noradrenaline. SP inhibited both electrically stimulated acetylcholine release and nicotinic release of norepinephrine (13). SP thus has both a presynaptic and a postsynaptic target.

For a more in-depth investigation of postsynaptic attack, studies were performed on isolated chromaffin cells with Bruce Livett (Melbourne) (14) to understand the modulation of synaptic transmission in these cells. This showed that SP has two effects. At first, it inhibits cholinergically induced catecholamine release, and second, it counteracts nicotine-induced desensitization of catecholamine release. Thus, SP can both inhibit excessive release and counteract too rapid depletion of release. Therefore, two separate points of attack are provided. Overall, SP is thus able to modulate synaptic transmission and act in the sense of the above-mentioned “Regulide” (15). It is the N-terminal tetrapeptide that inhibits both presynaptic acetylcholine release and postsynaptic norepinephrine release, thus modulating synaptic transmission in multiple ways. This is consistent with its role as an essential nucleus for the “anti-stress effect” of SP. These effects are independent of the NK 1 receptor. Apparently, the target for this modulation are the polyphosphoinositides (16).

### 2.4. Pharmacological effects on mast cells

Since it was known from the literature that SP can release histamine from peritoneal mast cells and that SP is released from sensory nerves upon antidromic stimulation, the Oehme group, in cooperation with the Pharmacological Institute of University College in London (UCL), began studies on modulation of synaptic transmission in mast cells. At first, SP, SP fragments and analogs were injected into the forearms in self-experiments. Later, volunteers from UCL joint the experiments. As expected, there was a dose-dependent redness and swelling on the forearms in these experiments. This was to be suppressed by antihistamines. N- and C-terminal SP fragments were ineffective. This implied that the entire SP molecule was necessary for histamine release from mast cells (17). Identical structure-activity relationships were shown on isolated peritoneal mast cells (18). These findings were considered significant for understanding the role of SP in the pathophysiology of inflammatory processes in various tissues, particularly in the bronchial tract.

Another study by Theoharides TC discusses the impact of the coronavirus (SARS-CoV-2) on the body, specifically focusing on the role of mast cells in the development of pulmonary symptoms and long-term complications in patients with COVID-19. The study suggested that activating mast cells can lead to the release of multiple proinflammatory cytokines, which can damage the lungs and contribute to pulmonary edema, inflammation, and thromboses. Additionally, it suggested that many patients who have recovered from or had mild symptoms of COVID-19 may experience diffuse, multiorgan symptoms months after the infection, similar to those presented by patients diagnosed with mast cell activation syndrome (MCAS). The study concluded that it is important to suspect, evaluate, and address MCAS in any patient with COVID-19 who experiences chronic multiorgan symptoms and suggests that blocking mast cells and their mediators, such as the natural flavonoid luteolin, could be useful in preventing and managing symptoms during the COVID-19 pandemic (19).

## 3. SP-actions in the respiratory tract

The action of SP in the respiratory tract played only a minor role at the SP symposia following the Stockholm SP conference. At the 1983 SP conference hosted by David Powell in Dublin, local release of SP in the bronchial tract of guinea pigs by various chemical irritants was reported (20). This SP release was associated with mucosal edema and bronchospasm. In 1984, the symposium on “Substance P—metabolism and biological actions,” initiated by Chris Jordan and Peter Oehme, in conjunction with the 9th IUPHAR International Congress of Pharmacology, was held in Maidstone (UK). In the review lecture by Bengt Pernow on “Substance P: present status and future prospects,” the function of SP in sensory nerves was discussed in detail. However, a crucial statement by Bengt Pernow was: “Although there is now strong evidence that SP is an important factor in the development of neurogenic inflammation, the mechanism by which SP exerts its biological effects is not clear” (21).

**Figure 2:**
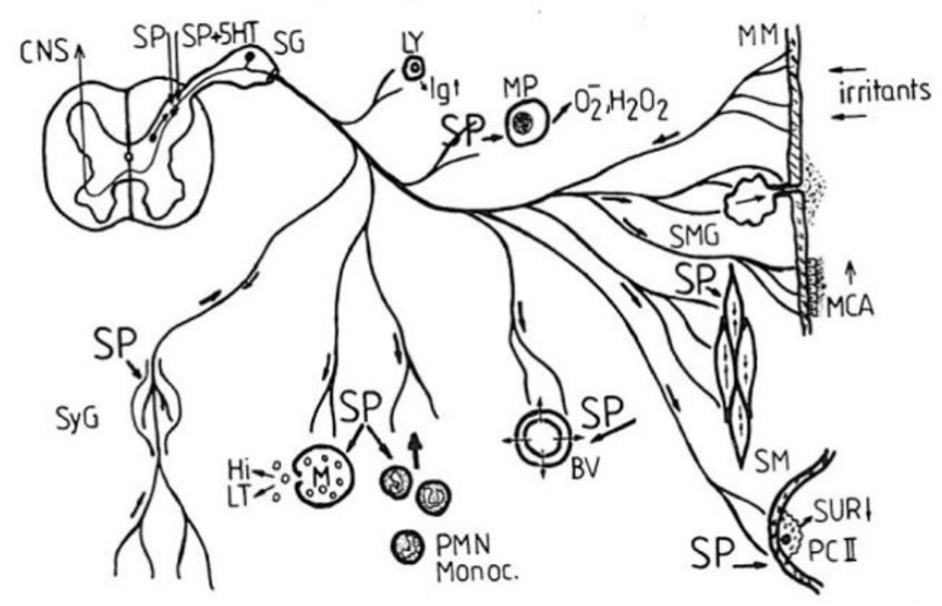
Proposed mechanism of Substance P in the respiratory tract (SP, spinal ganglion; Ly, lymphocyte; MP, macrophage; MM, mucous membrane; MCA, mucociliary activity; BV, blood vessels; SM, smooth musculature; SMG, mucous gland; PC II, pneumocyte type II; SUR, surfactant; SyG, sympathetic ganglion; Hi, histamine; LT, leukotrienes; Monoc., monocytes; PMN, polymorphonuclear neutrophils; CNS, central nervous system; 5-HT serotonin) (22).

Starting in 1987, Peter Oehme focused his group's work in this area and formed a joint working group with the Research Institute of Lung Disease and Tuberculosis in Berlin Buch. First of interest was the known bronchospastic effect of SP. As expected, SP1–11 showed a pronounced dose-dependent constrictor effect at the basal tone of the isolated guinea pig trachea (22). The C-terminal heptapeptide SP5–11 also caused a dose-dependent contraction of the isolated tracheal preparation. In contrast, the N-terminal tetrapeptide SP1–4 showed no constrictor effect. The contraction elicited by acetylcholine was significantly attenuated. Thus, the same picture emerged as in other pharmacological studies. The C-terminal has a direct effect; mediated *via* the NK 1 receptor. The N-terminal tetrapeptide has an indirect protective effect against the acetylcholine effect. This is mediated *via* a different target. Phosphatidylinositols have been discussed in this context. Figure 2 shows the mechanism of SP in the respiratory tract (22).

Since SP also acts on immunocompetent cells in the bronchial tract, it was of interest to determine whether differences also exist between the N- and C-terminal SP fragments. The studies were performed on spleen cell cultures from mice and mononuclear cells from rat lymph nodes (23). SP and the N-terminal sequences SP1–4 and SP1–7 were capable of secreting lymphokines with chemotactic properties for granulocytes and lymphocytes. The maximum of the dose-response curves was between 10–10 and 10–11, but the C-terminal fragments SP6–11, SP7–11, SP8–11, and SP9–11 were unable to induce lymphokines to be expressed.

Therefore, Oehme's group had planned to investigate both antagonists for the NK-1 receptor and N-terminal SP sequences for their therapeutic or preventive utility, primarily for the respiratory tract. In addition, capsaicin was of interest because of its influence on bronchial hyperreactivity (24). However, things were to turn out differently. With German unification, there were serious changes for both the Institute for Drug Research of the Academy of Sciences and the Research Institute for Lung Diseases and Tuberculosis. This led to the end of the research work on SP oriented on the bronchial tract in both institutes (25). A summary on pharmacological effects of SP can be found in the Sitzungsberichte der Akademie der Wissenschaften der DDR, newly published by de Gryuter Verlag (26), and in Reflections on Substance P-Research (27).

## 4. Role of SP in the first defense line of the respiratory tract

In 2021 saw the first contact between Peter Oehme and Riffat Mehboob. This was triggered by an event of the Leibniz Society of Sciences in Berlin with the chairman of the Drug Commission of the German Medical Association, Wolf-Dieter Ludwig, on the topic “What is the status of COVID-19” (28). An important statement of this meeting was that the next battle against Corona is to be fought in the respiratory tract. This statement prompted Peter Oehme to survey the literature in this direction. In doing so, Peter Oehme came across a paper by Riffat Mehboob on the importance of the NK 1 receptor in the therapy of COVID-19 (29). In particular, this work reported on the use of the NK-1 antagonist Aprepitant, in combination with dexamethasone, for the therapy of severe COVID courses. Riffat Mehboob proposed SP as a possible factor responsible for initiation of cytokine storming after getting infected with any foreign agent such as corona virus. Neurokinin-1 Receptor (NK-1R) antagonist, Aprepitant, was suggested as a potential drug for the treatment by inhibiting the receptor. Some evidences and commonalities were provided by her is support of this theory of SP involvement in respiratory tract infections including COVID-19 e.g., symptoms in COVID-19 infection and SP nociception, airway hypersensitivity/asthma in both phenomenon, variable patterns of COVID-19 disease severity in different age groups which is also addressed by SP theory, high death rate in COVID-19 patients having co-morbidities of diabetes, hypertension and cardiac disorders, viral load correlates with SP secretion and hence, its proposed that SP may be the trigger for cytokine storming during such inflammation. Aprepitant is NK-1R antagonist that has been approved for the treatment of chemotherapy-induced vomiting for a number of years (29).

A review by Karamyan VT suggested that inflammation was a major cause of complications from COVID-19, and studies had focused on pro-inflammatory cytokines and the “cytokine storm” as a mechanism to explain the severity of the disease. More recently in 2021, the article suggested that peptide bradykinin, its dysregulated signaling, or “bradykinin storm,” had emerged as a primary mechanism to explain COVID-19-related complications. The article also suggested that two closely related vasoactive peptides, SP and neurotensin, were also likely to have driven microvascular permeability and inflammation and been responsible for the development of COVID-19 pathology. It also postulated that in addition to ACE and neprilysin, peptidase neurolysin (Nln) was also likely to have contributed to accumulation of bradykinin, SP and neurotensin, and progression of the disease. In conclusion, it was proposed that “vasoactive peptide storm” may have underlain the severity of COVID-19 and that simultaneous inhibition of all three peptidic systems could have been therapeutically more advantageous than modulation of any single mechanism alone (30).

**Figure 3:**
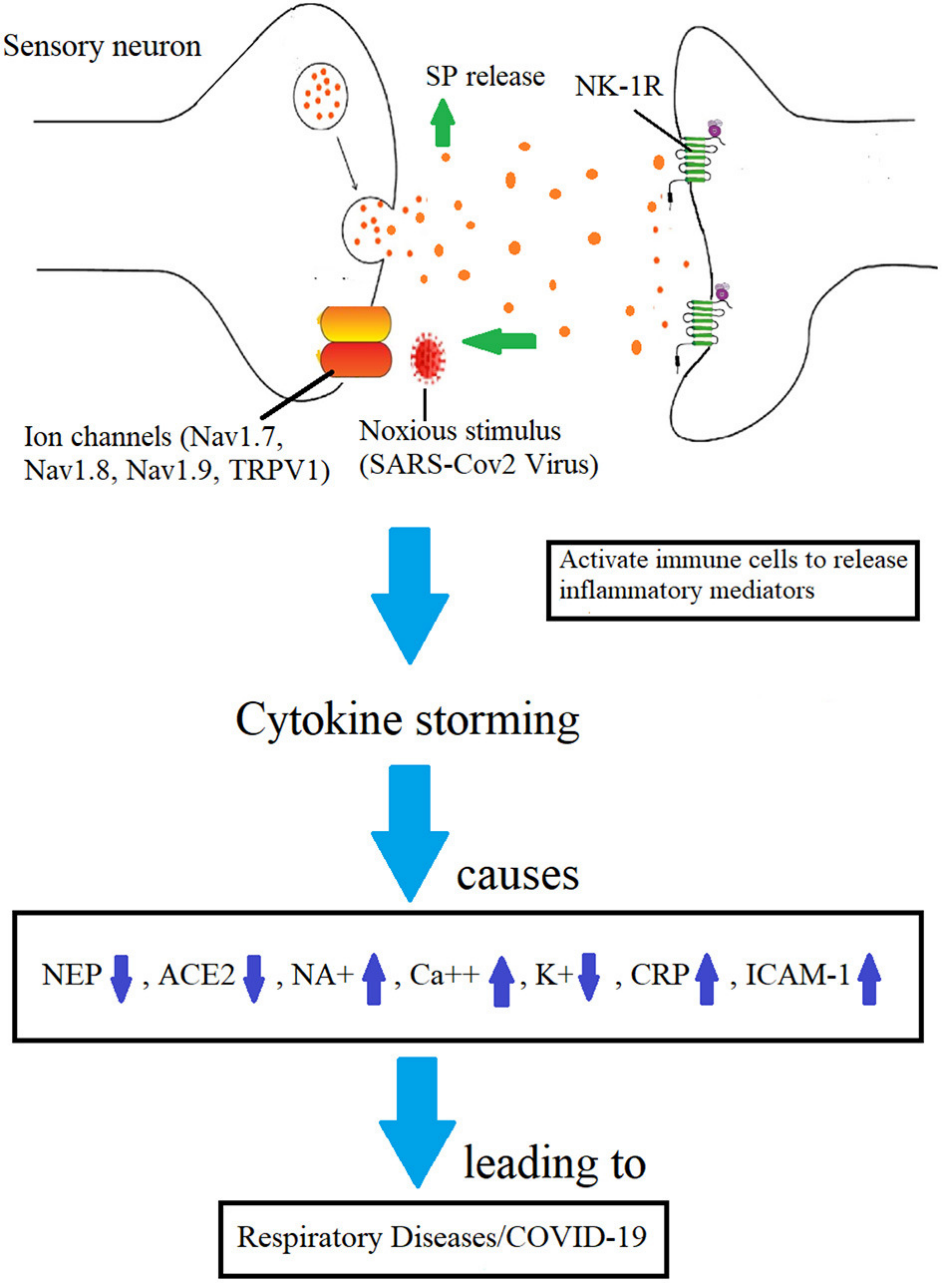
Mechanisms involved in the development of COVID-19 infection by which SP-induced inflammation is implicated. Increased BBB permeability and immune cell cytokine release are caused by SP's binding to the NK-1R on endothelial cells (28).

The immune reaction kills the virus to protect the host cells, but if it continues unchecked, it is known as cytokine storming, which may be lethal (Figures 1–3). Patients with COVID-19 infection may develop acute respiratory distress syndrome (ARDS) because immune cells continuously release inflammatory mediators (Figure 3). Therefore, the pathogen itself is not doing much damage, but cytokine storming is the main offender. Additionally, if restricted, illness severity could be reduced (31, 32). The immune system-stimulating effects of SP could cause a cytokine storm. The inflammatory pathways and, hence, the cytokine storming may both be stopped if its receptor is suppressed by aprepitant. When exposed to a toxic stimulus, SP is the first to react and acts as a quick defense mechanism to ensure survival. Comparing them to controls, NK-1R defective mice were shown to exhibit less pulmonary inflammation (33). Immune cells secrete SP, which has endocrine, paracrine, and autocrine effects (34). It can activate cells that are far away, such as smooth muscle cells, endothelium cells, lymphatics, white blood cells and fibroblasts. It interacts with NK-1R, stimulates the immunological and endocrine systems to produce inflammatory mediators in the airway tracts (35). It is also found on the cardio-ventilatory regulatory centers and phrenic nuclei, which regulate the diaphragm and respiration. It is concentrated in the brainstem nuclei that mediate respiratory regulation (36). Once formed, the SP/NK1-R complex starts a signaling chain that results in the production of IP3 and diacylglycerol (DAG) (37). The activation of NF-kB by macrophages and other immune cells results in the production of inflammatory mediators and the release of pro-inflammatory cytokines (38).

The study by Bellis et al., studied Neprilysin receptors in treating COVID-19. The study explained that SARS-CoV-2 disease causes ACE2 down-regulation and related decrease in angiotensin II degradation, which can lead to a “cytokine storm” and acute lung and cardiovascular injury. The researchers observed that current treatments, such as remdesivir and renin angiotensin system antagonists, have not been shown to be effective in reducing inflammation related to COVID-19. They suggested that neprilysin (NEP) may be an interesting target for preventing organ injury in COVID-19 patients, as it is involved in the degradation of molecules that prevent organ injury (39). NEP is involved in downregulation of SP and reduces inflammation (28) and supports the hypothesis discussed in this study.

The main symptom of COVID-19 is respiratory disease, but it is also becoming apparent that the disease affects multiple systems in the body, including the central nervous system (CNS) through the olfactory nerve and/or enteric nervous system. Neurological symptoms have been linked to a proinflammatory response in the CNS, caused by the ACE2 receptor being expressed in the brain, which ultimately leads to neuroinflammation. A study have shown that increased expression of TRPV1, a nonselective cation channel, leads to an increase in proinflammatory molecules such as substance P and IL-6, which are associated causing “cytokine storm” with more severe disease (40).

## 5. SP in ventilatory responses

SP has a major role in cardio-respiratory rhythm generation and control evidenced through previous study (35, 38) including ours conducted in University of Milan, Milan, Italy (41–44). They have an impact on how people react to ventilation since they are expressed in several brainstem regions. In a previous study, Riffat Mehboob and Anna Maria Lavezzi at the Lino Rossi Research Center, University of Milan, Italy, found that the increased expression of SP in the brainstem tissues of control infants as compared to infants who had experienced sudden infant death syndrome (SIDS), suggested that SP/NK-1R may be regulating the ventilatory regulation in newborns (41). In a related investigation, the brainstem nuclei of victims showed a marked reduction in SP and NK-1R binding. Due to a failure in cardiorespiratory regulation brought on by this altered SP expression, SIDS may result (45). In unexpected fetal fatalities, SP expression was increased (41, 43) and sudden adult death (46).

These findings may be correlated with mortalities in COVID-19 patients due to respiratory complications. SP also serves as a neuromodulator and vasodilator, contractions of smooth muscles in upper airways, increased excitatory potential by neurons, enhanced saliva production and a higher vascular permeability (38, 47). It may also lead to bronchoconstriction in pathological conditions (28, 47). Another study of Riffat Mehboob has discussed the fact that the gene encoding SP, TAC-1 has un-conventional networking properties such as it is singleton gene, has small protein interaction network and the members of tachykinin family have conserved aminoacyl sequences. These properties are responsible for vulnerability of TAC-1 gene and shows that it is a very important gene, any mutation in this gene may lead to fatal consequences as there will be no other gene copy to compensate its functions. These fatal outcomes may be sudden death due to respiratory failures. The other members of these gene pathway should also be explored (44).

SP and serotonin innervate the medullary motoneurons involved in upper respiratory tract (48) and laryngeal afferent system (38). In the bronchopulmonary C fibers of the respiratory tracts, SP, the most prevalent neuropeptide, and neurotransmitter, is found. It guards the lungs against any harm from irritating substances that are inhaled. The central nervous system (CNS) reacts to nociceptive stimuli by releasing nitric oxide, prostaglandins, and SP from the respiratory epithelium, as well as bronchoconstriction, cough, hypotension, sleep apnea, and mucus secretions in the lungs (48). NK-1R mRNA was found to be raised in broncho-alveolar lavage fluid (25), sputum samples (49) and lung tissue (50), in a study conducted on asthmatic patients. SP/NK-1R binding and the resulting interactions are also vital for the regulation of airway hyper responsiveness (AHR) (51).

An example of extreme hypersensitivity of the bronchial tract is SIDS when exposed to irritants, e.g., the cigarette smoke of the mother (41, 52). In this regard, immunohistochemical studies were published by Lavezzi et al. (41) and Mehboob et al. (42) in 2011 and 2017. These showed downregulated SP expression for SIDS-risk newborns in such brainstem areas that are important for respiratory regulation. This confirms earlier research by Oehme's group on infants at increased risk of SIDS, where a correlation between mean respiratory failure and low SP plasma levels was shown in the first 5 months of life. This has been discussed as an indication of delayed maturation of respiratory control mechanisms (45, 53). Vice versa, Mehboob and Lavezzi (43) questioned whether the minimal probability of healthy neonates and infants to become ill after corona infection is also related to the SP system. Fiona Bright from Australia discussed the abnormalities in the brainstem nuclei may be responsible for cardiorespiratory failure and hence SIDS (52, 54, 55). According to Mehboob et al., a fetus's brainstems exhibit very little SP expression. On the other hand, it is increased for newborns and lowered for kids and adults in controls. While the opposite results in unexpected fatalities (42).

## 6. SP/NK-1R, its relation to trigeminal ganglion, latency during corona virus infection

Another innovative idea for coronavirus latency during infection was put out by Riffat Mehboob. If we consider that the severe acute respiratory syndrome coronavirus 2 (SARS-CoV-2) virus is operating through the trigeminal ganglion (TG), which is the principal location for other latent viruses, she has highlighted the possibility of latency in SARS-CoV-2 virus infection. According to her, SP/NK-1R pathway is the key player in inflammation during COVID-19 infection as it may directly affect the ventilatory responses (48). The immune cells, along with other cells in the airways and the lung's epithelial lining, are impacted by the excessive secretion of SP by TG neurons (28). Corona virus may have a less unlikely chance of going latent and controlling the release of different TG peptides, including SP, by entering the TG *via* the trigeminal nerve in the eyes, nose, and mouth but the possibilities cannot be ruled out. The corona virus might be latent or quiescent in TG and could reactivate at any time. The patient could develop an infection as a result and experience no symptoms. After the initial infection, a virus' latency may be broken within the cell (56). Despite blood antibodies to the virus being present, the viral genome may stay in the host cell after primary infection and may be reactivated by any stressor (57).

The mesencephalic trigeminal nucleus and the TG in the brainstem both include some of the primary afferent neurons of the trigeminal nerve. The ocular (V1), maxillary (V2), and mandibular (V3) nerves are the three branches that make up the TrN. Each gives each of their distinct head regions innervation (58). The nociceptors, which are the free nerve ends of the trigeminal sensory afferents, are activated by pain or any other unpleasant stimulus, such as SARS-CoV2. These C-fiber sensory nerve fibers can be myelinated or not, and their cell bodies are found in the TG (42, 58). The trigeminal spinal caudalis (Vc) nucleus of the brainstem receives these impulses *via* afferent fibers. Here, they connect with the second order neurons that send signals to the thalamus and the limbic and somatosensory cortices. Trigeminal afferent neurons' activity can be altered by inflammation of orofacial tissues that the TrN innervates, leading to ectopic firing and increased sensitivity to painful stimuli. Numerous mediators, including neurotrophic factors or neuropeptides at nerve ends, such SP, CGRP, and serotonin, induce sensitization. TG and TrN's SP and CGRP levels rise in response to painful stimuli, including nerve damage (59). Nicotinic stimulation of polyphosphoinositide turnover in rat adrenal medulla slices was studied by Minenko et al. (60) and the influence of adrenal demedullation on stress-related behavior in wistar rats was investigated by Roskte et al. (61), in the same year. One of the stress related parameters measured was SP in addition to blood pressure, pain sensitivity, endogenous opiod system etc.

A study by Henri et al., used computational methods to identify potential drug candidates that can bind to the nucleoprotein N of SARS-CoV-2, the virus that causes COVID-19. The researchers used a new model of N, which was built using an existing model and refined by molecular dynamics simulations. The predicted drug candidates were neuropeptides, such as substance P (1–7) and enkephalin, which bind to a large site on the C-terminal or N-terminal β-sheet of N. The study also found that some variants of N, such as BA4 and BA5, also have large binding sites. The binding sites of the predicted drug candidates were then tested using surface plasmon resonance experiments. The study found that the drugs likely impede RNA binding to N, which could inhibit viral replication. The study suggested that these neuropeptides may play a role in the symptoms of long COVID-19 and that drugs targeting N may help reduce the risk of brain fog and stroke. This link of neuropeptides involved in COVID-19 supports our hypothesis (62).

## 7. Neurologic manifestations post COVID-19 and role of SP

Clinical manifestations of COVID-19 are variable and hence the neurological symptoms such as anosmia, ageusia, central hemorrhage, infarction also vary depending on age and comorbidities (63). Patients with long COVID-19 infection may experience post-intensive care syndrome, post-viral fatigue syndrome, permanent organ damage and long COVID syndrome (64, 65). Central nervous system disorders in COVID-19 are more than anticipated so far. Peripheral nerves and skeletal muscles are affected to a lesser extent. In majority of the cases, there is no direct attack of the virus toward vulnerable structures, explaining the possibility that why nervous system manifestations manifest favorably to immune suppression (66).

SP is also involved in post COVID olfactory dysfunction. Schirinzi et al., research study investigated the activity of two inflammatory pathways, SP and Prokineticin-2 (PK2), within the olfactory neurons (ONs) of patients to understand the mechanisms of persistent olfactory dysfunction (OD) post-COVID-19. The study collected ONs from 10 patients with persistent post-COVID-19 OD and 10 healthy controls using non-invasive brushing. Gene expression levels of SP, Neurokinin receptor 1, Interleukin-1β (IL-1β), PK2, PK2 receptors type 1 and 2, and Prokineticin-2-long peptide were measured in ONs by Real Time-PCR and correlated with residual olfaction. Immunofluorescence staining was also performed to quantify SP and PK2 proteins. The results showed that patients with OD had increased levels of both SP and PK2 in ONs compared to healthy controls, with the latter being proportional to residual olfaction. This study provides preliminary evidence that both SP and PK2 pathways may have a role in persistent post-COVID-19 OD. The sustained activation of SP, lasting months after infection's resolution, might foster chronic inflammation and contribute to hyposmia, while the PK2 expression could instead support the smell recovery (67).

As the research on COVID-19 infections continued to evolve, various possible meachnisms of virus attack on CNS was suggested. One such mechanism was angiotensin-converting-enzyme-2 receptor as a potential modulator of coronavirus related CNS damage and suggested that it damages the cerebrovascular endothelium and brain parenchyma, the latter predominantly in the medial temporal lobe, resulting in apoptosis and necrosis (68). Neurons and glial cells express ACE2 receptors in the CNS, and recent studies suggest that activated glial cells contribute to neuroinflammation and the devastating effects of SARS-CoV-2 infection on the CNS. The SARS-CoV-2-induced immune-mediated demyelinating disease, cerebrovascular damage, neurodegeneration, and depression are some of the neurological complications (69). We have also proposed a novel theory that coronavirus may stimulate nociceptive pathways after entering the trigeminal ganglion of the brainstem where it trigers the release of SP. SP binds to Neurokinin-1 Receptor and initiate cytokine storming in lungs leading to complications related to COVID-19 infection. Virus may also become latent in trigeminal ganglion (70).

Evidence of possible routes of SARS-CoV-2 neuroinvasion through systemic circulation and crossing the blood-brain barrier making its way to the central nervous system is still lacking (71). Pathophysiology and neurological manifestations of COVID-19 post infection was discussed by Bobker et al. with focus on headache. Many variations in the neurological symptoms of patients were observed and more researches were suggested for better understanding (71). Persistent post-COVID-19 OD is an unknown syndrome that could lead to neurological complications. Because of the potential long-term neurological consequences, persistent olfactory dysfunction (OD) is one of the most common and concerning problems of long-term COVID-19. OD patients had higher amounts of SP than controls. There is preliminary evidence that SP pathways may play a role in chronic post COVID-19 OD, making both of them potential therapeutic targets (72).

## 8. Future aspects

The conclusion in one of our previous paper (28), “actually it is not the virus that is fatal and causing mortalities, but the cytokine storming activated and initiated by SP is bringing the disaster,” we believe this situation with COVID-19 could be explained by the historical views of Rudolf Virchow (1821–1902), Robert Koch (1843–1910), Max von Pettenkofer (1818–1901), and Oscar Liebreich (1839–1908) on the proper control of epidemics (26, 73). At the end of these discussions, in conjunction with the cholera epidemics of the time, was the statement that the germ is not the disease, but that disease germ, vector, and human mutually influence each other and must, therefore, be considered equally (74).

While contaminated water was the main vector for the cholera epidemics in former times, air is the main vector for the corona pandemics today. The air vector is certainly a multi-layered problem. In addition to viruses as pathogens, the air today contains a large number of pollutants that must be taken seriously. It is significant for the further scientific work that air and respiratory tract are closely related. The findings presented in this paper show that the neuropeptide SP has a defense function in the respiratory tract (75).

At this point, here are some perspective thoughts connected with the goal of linking Substance P research more closely with research on corona diseases. A first thought is that the viruses (or pollutants) entering *via* the respiratory tract are to be understood as stressors. The respiratory tract has the task to recognize these stressors as such to organize the local defense, to impede or block further penetration and, if possible, to destroy the stressors. In this context, the respective state of the immune system is certainly decisive for the subsequent outcome. From the findings of Mehboob et al. (28, 43, 47, 72), and the Oehme group (45, 53), it is clear that the different infection rates for COVID-19 infections or the frequencies of sudden death infant syndromes correlate with the SP plasma level. In addition, from the experience of the current corona epidemic, children are equally likely to become infected with corona but are much less likely to develop corona than adults. In addition, the extensive studies by Oehme and Hecht (see Section 2.2) on experimental animals and humans show that there is a clear relationship between stress sensitivity and SP levels: Low SP levels = high stress sensitivity (12, 28). It would be useful to follow up on these findings and investigate in adult Corona-infected individuals whether the frequency of transition from infection to disease correlates with SP levels in plasma or bronchial lavage.

A second thought: it has been demonstrated by Mehboob et al. (28, 76) for Aprepitant that this SP antagonist, in combination with dexamethasone, improves symptomatology in severe corona course. This finding is explained by a reduction in cytokine storming triggered by SP in the deeper pulmonary alveoli. However, the primary response of coronavirus occurs in the upper nasopharynx. Here, SP (antidrome) is also released to trigger defense processes. Since these local processes determine to a large extent the further course of the infection, they should be investigated in depth. Of particular interest would be whether N-terminal dipeptides, especially Lys-Pro, are cleaved from SP1–11 by enzymatic cleavage during this local SP release. For this dipeptide, both stress-protective effects (see Sections 2.2 and 2.3) and positive effects in the respiratory tract (see Section 3) have been demonstrated. In addition, Lys-Pro was found to stimulate nerve fiber growth in tissue culture (74). Therefore, a Lys-Pro derivative was also applied for a patent with the indication “wound healing” (77).

Drug repurposing, the process of identifying existing drugs that can be used to treat new conditions, has several potential benefits for COVID-19 treatment development. These include shorter development time, reduced costs, and faster regulatory approval. A study by Egieyeh et al., used computational methods to predict drugs from the Drug Bank that may bind to the SARS-CoV-2 spike protein on the human ACE2 receptor and inhibit the protein-protein interaction required for infection. The predicted drugs, which include peptide-based drugs like Sar9 Met (O2)11-Substance P and BV2, may have potential for treating COVID-19 and have already been investigated for other indications such as ARDS and viral infections. The study also explored the current and proposed pharmacological uses of the predicted drugs, finding that some have been investigated for treatment of acute respiratory distress syndrome (ARDS), viral infection, inflammation, and angioedema, as well as stimulation of the immune system and enhancement of antiviral agents against influenza virus. Similar computational study can also be performed on SP/NK1R to test its potential in treating COVID-19 (78).

To prevent and contain this epidemic, it is imperative that new medicinal approaches be developed. A novel class of medications called NK-1R antagonists has antidepressant, antiemetic, and anxiolytic effects. Aprepitant, Rolapitant, Casopitant, Netupitant, Maropitant, and Fosaprepitant are a few examples of NK-1R inhibitors (79). In 2003, the FDA approved aprepitant as the first NK-1R antagonist (80). It could be a part of a viral respiratory disease therapy plan. In a phase 2 trial (VOLCANO-1) for the treatment of persistent refractory cough, orvepitants had dramatically reduced the symptoms (81). It is well established that NK-1R antagonists have an anti-inflammatory effect on rats, and that SP and NK-1Rs are both increased during the inflammatory processes (82). It may be advantageous to pharmacologically suppress SP-signaling in COVID-19 infection. The use of NK-1R antagonists may be advised to alleviate SP-related symptoms. In patients with viral myocarditis, SP-receptor antagonism may also be suggested as a treatment approach (83). Riffat Mehboob and her team just completed a randomized clinical trial in which they saw very encouraging patient outcomes for COVID-19 treatment. There were two arms; one received standard treatment and the other received the NK-1R antagonist, aprepitant, in addition. Both groups also received dexamethasone treatment. 52 patients were placed in control group A and 67 patients were placed in intervention group B out of a total 119 patients who were randomly assigned to both of these arms. Before and after the intervention, blood parameters examined in both groups. Patients who received a combination of aprepitant and dexamethasone medication demonstrated improved clinical results, laboratory findings, and decreased levels of the inflammatory marker C-reactive protein (76). Here, we propose that the pathogenesis of COVID-19 infection brought on by SARS-CoV-2 is mediated by SP/NK-1R. As in other airway infections, it might be brought on by cytokine storming exacerbating the inflammatory pathways. Corticosteroids, antibiotics, purified intravenous immunoglobulins, and anti-cytokine therapy should all be used together as part of the suggested treatment plan (84). Overall, further contact between SP and corona research would be enlightening and could promote greater collaboration between environmental and medical research.

## 9. Conclusions

SP release from trigeminal nerve as a consequence of a nociceptive stimulus is directly related to the respiratory complications in COVID-19 and other respiratory illnesses. It causes an increased inflammation and must be blocked by using Aprepitant, a neurokinin 1 receptor antagonist along with glucocorticoid, dexamethasone. Dexamethasone will activate the enzyme neutral endopeptidase which is responsible for degradation of SP and Aprepitant may block the NK-1R. Hence, the cytokine storm will be inhibited by blocking this pathway and the disease progression too. This therapeutic strategy may be effective as a successful clinical trial was conducted on COVID-19 patients in Pakistan by Riffat Mehboob and her team (76). These findings urge for more investigation on this drug, further clinical trials in other countries and a drug should be formulated based on this strategy. It will be a new and effective treatment for COVID-19.

Overall, this summarized review urges further research into this magical regulatory peptide (regulide) which can be used as treatment strategy for various diseases by maintaining an optimal balance and regulation of this peptide within plasma. We can foresee future of treatments into peptides and peptide researches and should be investigated on priority basis.

## Data availability statement

The original contributions presented in the study are included in the article/supplementary material, further inquiries can be directed to the corresponding authors.

## Author contributions

All authors listed have made a substantial, direct, and intellectual contribution to the work and approved it for publication.
